# Components of the Canonical and Non-Canonical Wnt Pathways Are Not Mis-Expressed in Pituitary Tumors

**DOI:** 10.1371/journal.pone.0062424

**Published:** 2013-04-26

**Authors:** Leandro Machado Colli, Fabiano Saggioro, Luciano Neder Serafini, Renata Costa Camargo, Helio Rubens Machado, Ayrton Custodio Moreira, Sonir R. Antonini, Margaret de Castro

**Affiliations:** 1 Department of Internal Medicine, School of Medicine of Ribeirao Preto, University of Sao Paulo, Sao Paulo, Brazil; 2 Department of Pathology, School of Medicine of Ribeirao Preto, University of Sao Paulo, Sao Paulo, Brazil; 3 Department of Surgery, School of Medicine of Ribeirao Preto, University of Sao Paulo, Sao Paulo, Brazil; 4 Department of Pediatrics, School of Medicine of Ribeirao Preto, University of Sao Paulo, Sao Paulo, Brazil; University of Cordoba, Spain

## Abstract

**Introduction:**

Canonical and non-canonical Wnt pathways are involved in the genesis of multiple tumors; however, their role in pituitary tumorigenesis is mostly unknown.

**Objective:**

This study evaluated gene and protein expression of Wnt pathways in pituitary tumors and whether these expression correlate to clinical outcome.

**Materials and Methods:**

Genes of the Wnt canonical pathway: activating ligands (*WNT11, WNT4, WNT5A*), binding inhibitors (*DKK3, sFRP1*), β-catenin (*CTNNB1*), β-catenin degradation complex (*APC, AXIN1, GSK3β*), inhibitor of β-catenin degradation complex (*AKT1*), sequester of β-catenin (*CDH1*), pathway effectors (*TCF7, MAPK8, NFAT5*), pathway mediators (*DVL-1, DVL-2, DVL-3, PRICKLE, VANGL1*), target genes (*MYB, MYC, WISP2, SPRY1, TP53, CCND1*); calcium dependent pathway (*PLCB1, CAMK2A, PRKCA, CHP*); and planar cell polarity pathway (*PTK7, DAAM1, RHOA*) were evaluated by QPCR, in 19 GH-, 18 ACTH-secreting, 21 non-secreting (NS) pituitary tumors, and 5 normal pituitaries. Also, the main effectors of canonical (β-catenin), planar cell polarity (JNK), and calcium dependent (NFAT5) Wnt pathways were evaluated by immunohistochemistry.

**Results:**

There are no differences in gene expression of canonical and non-canonical Wnt pathways between all studied subtypes of pituitary tumors and normal pituitaries, except for *WISP2,* which was over-expressed in ACTH-secreting tumors compared to normal pituitaries (4.8x; p = 0.02), NS pituitary tumors (7.7x; p = 0.004) and GH-secreting tumors (5.0x; p = 0.05). β-catenin, NFAT5 and JNK proteins showed no expression in normal pituitaries and in any of the pituitary tumor subtypes. Furthermore, no association of the studied gene or protein expression was observed with tumor size, recurrence, and progressive disease. The hierarchical clustering showed a regular pattern of genes of the canonical and non-canonical Wnt pathways randomly distributed throughout the dendrogram.

**Conclusions:**

Our data reinforce previous reports suggesting no activation of canonical Wnt pathway in pituitary tumorigenesis. Moreover, we describe, for the first time, evidence that non-canonical Wnt pathways are also not mis-expressed in the pituitary tumors.

## Introduction

Pituitary tumors cause significant morbidity by compression of central nervous system structures or inappropriate expression of pituitary hormones [1]. The molecular pathogenesis of sporadic or familial pituitary tumors remains mostly unknown; although, studies on genetic syndromes have provided new insights into the molecular basis of these tumors [1]–[6].

The Wnt pathway influences embryonic development, including axial disposition, organ formation, cell fate, and self-renewal of stem cells [7]. In physiological conditions, the Wnt pathway is activated when a Wnt ligand binds to its cell-surface receptor [8]. Ligand binding results in the dissociation of the β-catenin cytoplasmic degradation complex, comprising GSK-3β, APC, and AXIN1, resulting in β-catenin phosphorylation inhibition [7]. β-catenin thereby accumulates in the cytoplasm, translocates to the nucleus, and by binding to TCF/LEF, regulates the expression of several Wnt target genes, involved in cell growth and differentiation [8].

The Wnt signaling pathway has been implicated in the pathogenesis of several tumor types, such as colorectal [9], pediatric adrenocortical tumors [10], and craniopharyngioma [11]. In this latter, almost 80% of the cases of the adamantinomatous craniopharyngioma type showed aberrant cytoplasm and nucleus β-catenin accumulation in contrary to the classical concentration in the cell membrane observed in normal tissue [12]. In addition, the prevalence of *CTNNB1* mutations in craniopharyngiomas observed in different series varies from 16 to 100% [11]–[14]. On the other hand, the role of Wnt pathway in pituitary tumors is still controversial in the literature. The initial work revealed nuclear β-catenin accumulation and suggested the involvement of the canonical pathway in pituitary tumorigenesis [15]. However, other studies failed to confirm the nuclear expression of β-catenin in large series of pituitary tumors [12], [16], [17].

Besides the canonical Wnt pathway, in which β-catenin is the central effector; there are the calcium-dependent and the planar cell polarity non-canonical Wnt pathways, which are β-catenin independent. Wnt binding to frizzled receptors signals to cell polarity and migration mediated by Disheveled (DVL) and JNK and to cell migration and invasion through stimulated calcium flux and activation of calcium-dependent enzymes calcium/calmodulin-dependent kinase II (CAMKII), calpain, and PKC [18], [19]. Wnt can also signal in a β-catenin–independent fashion by binding to non-Frizzled receptors such as ROR2 [20]. There are relatively little understanding of the roles and the mechanisms of non-canonical Wnt pathways in tumorigenesis. Previous studies have implicated these pathways in cancer development [21]–[23]. Over-expression of Wnt5a is associated with migration and invasiveness in several cancers, including gastric and pancreatic as well as melanoma [24]–26. There are lacking studies addressing the involvement of the non-canonical Wnt pathways in the pathogenesis of the pituitary tumors.

In this context, the present study evaluates gene expression and the main effector proteins of canonical and non-canonical Wnt pathways in ACTH-, GH- secreting and non-secreting pituitary tumors to clarify their putative involvement in the pituitary tumorigenesis.

## Materials and Methods

The study was approved by the Institutional Review Board of the University Hospital of the School of Medicine of Ribeirao Preto, University of Sao Paulo, Brazil (Process n° 8334/2005, 3608/2006, and 5283/2007). All participants gave written informed consent.

We studied 58 patients presenting pituitary tumors: 18 ACTH-, 19 GH-secreting and 21 non-secreting tumors. Table S1, Table S2, Table S3 show individual clinical and laboratorial features of the patients with different types of pituitary tumors.

All pituitary tumor samples were collected during transsphenoidal surgery. Part of the tumor tissue was paraffin embedded for routine histopathological examination and the other part was microdissected by experienced pathologist to separate tumoral from non-tumoral tissues. The microdissected tumor tissue was disrupted using a Polytron™ homogenizer and kept at −70°C for molecular studies. The control group comprises five normal pituitary tissues obtained during autopsies from subjects who had died due to cardiovascular acute disease, without previous evidence of any endocrine disease.

Tumoral total RNA were isolated by TRIzol® reagent (Invitrogen Life Technologies, Carlsbad, CA). Sample integrity was evaluated by spectrophotometry at an absorbance of 260/280 nm and by agarose gel electrophoresis. cDNA were obtained using High capacity cDNA Reverse Transcription kit (Applied Biosystems, Foster City, CA, USA).

The relative expression of some genes of the Canonical Wnt pathway (*WNT11, WNT4, WNT5A, DKK3, sFRP1, CTNNB1, APC, AXIN1, GSK3β, AKT1, CDH1, and TCF7*), Calcium/Wnt pathway (*PLCB1, CAMK2A, PRKCA, CHP, and NFAT5*), and Planar cell polarity pathway (*PRICKLE, VANGL1, DVL-1, DVL-2, DVL-3, PTK7, DAAM1, RHOA, and MAPK8*), as well as the target genes (*MYB, MYC, WISP2, SPRY1, TP53, and CCND1*), and endogenous controls (*GUSβ, TBP,* and *PGK1*) were performed byTaqMan® Real Time PCR Assay (Applied Biosystems, Foster City, CA, USA). The specific probes and assay IDs are presented in Table S4. These genes were selected by literature reviews and by the KEGG pathway [7], [27], [28]. Reactions were incubated in a 96-well optical plate at 95°C for 10min, followed by 40 cycles of 95°C for 15sec and 60°C for 1 min. Gene expression was calculated by QPCR software [29], using efficiency of each reaction. Fold change was determined by the median of each gene expression observed in pituitary tumors related to the median of gene expression observed in normal pituitaries.

Immunohistochemistry (IHC) studies were performed in tumor samples after deparaffinization, rehydration, antigen retrieval in citrate buffer solution (pH 6.0) for 45 min, and incubation for 2 h at room temperature with primary antibodies for the main effectors of canonical (β-catenin - Santa Cruz SC-7963), planar cell polarity (JNK - Abcam AB18680) and Calcium dependent (NFAT5 - Abcam AB3446) Wnt pathways. In ACTH-secreting pituitary tumors, IHC was performed in nine of the 18 samples studied by qPCR and in seven other ACTH-pituitary secreting tumors. The avidin-biotin system (Vectastain Elite ABC Kit, Burlingame, CA) were used to signal detection. We used craniopharyngioma tissue as positive control for β-catenin antibody; for NFAT5 and JNK antibodies we used two positive samples from GH-secreting pituitary tumor tissues. Each sample was assigned in 10 random fields as percentage of stained cells: negative (less than 1%), +1 (weak, 1–10%), +2 (moderate, 10–50%), and +3 (strong, more than 50%).

The expression of each gene in pituitary tumors and in normal pituitary samples is presented as mean, standard deviation, median, and range. Statistical analysis was performed using Mann-Whitney test or Kruskal-Wallis test for multiple continuous variables (post test of Nemeyi-Dunn), when appropriate. Multivariable regression was used for continuous variables and logistic regression for categorical variables, such as gender, age at diagnosis, tumor size, altered visual field, remission of the disease, and tumor progression. Fisher’s exact test was also used for comparison of categorical variables, such as staining intensity by IHC between tumors and normal pituitaries. We used Agnes method for hierarchical clustering for gene expression in each tumor subtype. Data were analyzed by the R Project Software [30]. Differences were considered significant at p<0.05.

## Results

Table1 shows the expression of studied genes of the canonical and non-canonical Wnt pathways in ACTH-, GH-secreting, and non-secreting pituitary tumors, and also in normal pituitaries. There are no differences in the expression of canonical and non-canonical Wnt pathway genes between each pituitary tumor subtype and normal pituitaries, as well as among all pituitary tumor subtypes, except for *WISP2* which was over-expressed in ACTH-secreting tumors compared to normal pituitaries (4.8x; p = 0.02), to non-secreting pituitary tumors (7.7x; p = 0.004) and to GH-secreting tumors (5.0x, p = 0.05).

**Table 1:** Wnt Pathway gene expression in Pituitary Tumors.

Gene	ACTH-Secreting Tumor	GH-Secreting Tumor	Non-Secreting Tumor	Normal Pituitary	p-value Kruskal-Wallis
	Mean ± SD	Mean ± SD	Mean ± SD	Mean ± SD	
	median (range)	median (range)	median (range)	median (range)	
***AKT1***	1.45±1.38	1.13±0.43	1.21±0.73	0.85±0.23	0.84
	0.88 (0.18–5.78)	1.19 (0.49–1.77)	0.84 (0.36–2.74)	0.88 (0.50–1.10)	
***APC***	1.25±0.91	1.18±0.75	1.30±1.00	1.35±0.93	0.99
	1.02 (0.17–3.17)	1.20 (0.42–3.62)	0.98 (0.09–3.07)	1.13 (0.68–2.94)	
***AXIN1***	1.16±0.43	1.34±1.51	1.35±1.39	0.97±0.29	0.58
	1.13 (0.46–2.32)	0.86 (0.38–5.95)	0.85 (0.18–6.54)	0.91 (0.56–1.30)	
***CAMK2A***	3.15±4.93	1.67±1.55	4.34±9.16	1.38±1.61	0.97
	1.08 (0.05–16.93)	1.06 (0.04–4.68)	0.99 (0.01–41.95)	0.63 (0.30–4.21)	
***CCND1***	0.46±0.42	0.79±10.66	0.79±0.95	1.01±0.41	0.43
	0.42 (0.05–1.46)	0.57 (0.04–2.16)	0.45 (0.04–3.85)	0.94 (0.50–1.65)	
***CDH1***	1.60±1.61	2.74±2.17	1.56±1.46	0.74±0.16	0.24
	1.18 (0.07–6.05)	3.69 (0.01–7.43)	0.95 (0.05–5.69)	0.70 (0.33–1.13 )	
***CHP***	1.24±0.96	1.22±0.70	1.17±0.73	0.96±0.16	0.97
	1.08 (0.08–4.11)	0.95 (0.44–3.1)	1.06 (0.32–3.07)	0.95 (0.78–1.21)	
***CTNNB1***	1.22±0.79	1.09±0.58	1.17±0.83	0.84±0.42	0.72
	1.02 (0.34–3.98)	0.89 (0.43–2.63)	0.91 (0.33–3.57)	0.64 (0.39–1.42)	
***DAAM1***	1.29±0.79	1.67±2.16	1.91±2.88	1.02±0.25	0.54
	1.09 (0.17–3.37)	0.92 (0.23–8.97)	0.78 (0.10–11.64)	0.98 (0.78–1.41)	
***DKK3***	1.21±0.69	1.94±2.32	1.64±2.12	2.56±1.42	0.24
	1.16 (0.10–2.45)	1.03 (0.05–7,56)	0.66 (0.11–7.09)	2.1 (1.13–4.34)	
***DVL-1***	1.07±0.49	1.26±0.63	1.18±0.87	0.86±0.41	0.59
	0.99 (0.52–2.10)	1.07 (0.3–2.59)	0.99 (0.40–3.72)	0.68 (0.43–1.46)	
***DVL-2***	1.15±0.55	1.18±0.50	1.32±1.01	0.92±0.29	0.78
	1.11 (0.41–2.41)	1.13 (0.38–1.96)	1.00 (0.42–3.71)	0.91 (0.51–1.28)	
***DVL-3***	0.99±0.31	1.18±0.43	1.17±0.77	0.81±0.37	0.29
	0.95 (0.64–1.88)	1.15 (0.56–1.96)	0.91 (0.46–3.74)	0.60 (0.45–1.25)	
***GSK3β***	1.12±0.49	1.10±0.42	1.21±0.92	0.97±0.08	0.76
	1.06 (0.39–2.52)	1.05 (0.40–1.83)	0.86 (0.33–3.50)	0.99 (0.85–1.04)	
***MAPK8***	1.30±1.76	1.17±0.57	1.34±1.15	0.98±0.22	0.34
	0.63 (0.18–7.32)	1.09 (0.39–2.42)	0.96 (0.28–5.14)	0.86 (0.77–1.26)	
***MYB***	2.02±4.17	0.74±0.95	7.82±32.11	0.81±0.39	0.56
	0.47 (0.10–17.69)	0.28 (0–3.68)	0.50 (0.00–147.89)	0.66 (0.42–1.31)	
***MYC***	1.80±2.30	1.58±1.35	2.35±4.64	1.20±1.10	0.93
	0.97 (0.17–8.31)	1.04 (0.11–4.90)	1.05 (0.06–21.48)	0.51 (0.31–2.76)	
***NFAT5***	1.16±0.62	1.18±0.42	1.29±0.89	1.24±0.32	0.95
	1.03 (0.24–2.45)	1.17 (0.61–2.0)	1.28 (0.18–3.49)	1.12 (0.94–1.72)	
***PLCB1***	1.71±1.96	1.26±0.70	1.89±2.80	0.92±0.27	0.90
	1.29 (0.06–8.46)	1.27 (0.32–3.55)	0.88 (0.06–12.60)	0.88 (0.53–1.23)	
***PRICKLE***	1.37±1.06	1.39±1.03	1.40±1.19	0.91±0.38	0.91
	1.18 (0.22–4.32)	0.99 (0.18–4.09)	0.88 (0.08–3.85)	0.78 (0.61–1.54)	
***PRKCA***	1.50±1.45	1.31±0.76	1.47±2.01	1.01±0.16	0.82
	0.87 (0.27–6.29)	1.14 (0.07–2.69)	1.00 (0.25–9.64)	0.93 (0.87–1.24)	
***PTK7***	1.84±2.77	1.45±0.87	1.24±0.63	0.93±0.29	0.64
	1.17 (0.15–12.36)	1.51 (0.05–3.31)	1.21 (0.13–2.36)	0.96 (0.53–1.29)	
***RHOA***	1.05±0.32	1.13±0.43	1.08±0.45	0.98±0.23	0.93
	1.06 (0.61–1.64)	1.07 (0.44–1.82)	0.95 (0.41–1.94)	1.04 (0.72–1.29)	
***SPRY1***	57.55±234.97	1.52±1.45	1.61±1.65	0.84±0.38	0.83
	1.02 (0.13–999.0)	1.12 (0.12–5.71)	1.04 (0.18–5.99)	0.87 (0.35–1.25)	
***TCF7***	1.86±2.55	1.66±2.17	1.38±1.48	4.97±5.71	0.31
	0.94 (0.08–10.35)	0.95 (0.20–9.93)	0.75 (0.07–4.61)	3.49 (0.28–14.58)	
***TP53***	1.21±0.69	1.33±1.07	1.16±0.91	1.19±0.44	0.71
	1.12 (0.06–2.80)	1.04 (0.26–4.4)	0.86 (0.30–4.11)	1.24 (0.65–1.83)	
***VANGL1***	1.28±0.96	1.32±1.52	3.01±5.83	0.99±0.30	0.94
	1.15 (0.27–3.96)	0.71 (0.14–7.05)	0.87 (0.10–24.23)	1.09 (0.49–1.26)	
***WISP2***	**1.34±1.34**	**2.08±5.42**	**0.21±0.18**	**0.36±0.44**	**0.01**
	**1.02 (0.03–4.59)**	**0.21 (0–23.77)**	**0.14 (0.00–0.56)**	**0.20 (0.07–1.13)**	
***Wnt11***	1.85±2.46	4.16±7.82	1.69±1.52	0.82±0.13	0.91
	1.03 (0.07–9.99)	0.45 (0.03–26.78)	1.37 (0.08–5.57)	0.77 (0.71–1.03)	
***Wnt4***	2.24±2.67	2.46±3.36	2.44±3.83	3.55±3.54	0.31
	1.27 (0.14–11.18)	0.79 (0.06–11.2)	0.83 (0.01–14.92)	1.5 (1.11–9.25)	
***Wnt5A***	1.99±2.30	1.43±1.24	3.27±6.64	1.55±1.20	0.76
	1.03 (0.04–7.92)	0.85 (0.30–4.43)	0.74 (0.04–24.23)	1.24 (0.61–3.62)	
***sFRP1***	3.23±3.23	1.95±3.74	14.25±39.05	0.50±0.41	0.30
	2.46 (0.05–11.06)	0.23 (0.01–11.77)	0.73 (0.01–179.33)	0.50 (0.17–1.14)	
***GUSβ***	1.01±0.35	1.26±0.487	1.14±0.80	1.04±0.24	0.59
	1.06 (0.41–1.72)	1.33 (0.51–2.02)	0.94 (0.50–4.26)	1.07 (0.68–1.35)	
***PGK1***	1.13±0.45	0.91±0.30	1.19±0.78	0.94±0.17	0.98
	1.06 (0.46–1.88)	0.84 (0.44–1.44)	0.98 (0.38–3.88)	0.96 (0.69–1.13)	
***TBP***	1.05±0.25	1.06±0.34	1.04±0.30	1.12±0.28	0.99
	1.08 (0.50–1.39)	1.02 (0.49–1.65)	1.00 (0.44–1.63)	1.07 (0.68–1.54)	

There was no association between the expression of each studied gene and gender, age at diagnosis, tumor size, altered visual field, remission of the disease, or tumor progression in any subtype of the pituitary tumors, but *VANGL1* which was positively associated with bigger tumors (p = 0.04; logistic regression) in all subtypes of pituitary tumors. Using logistic regression analysis, non-secreting pituitary tumors, as a group, were also associated with bigger tumor size; these tumors have the chance to be larger than ACTH- (+2.3 cm; p = 0.03) and GH-secreting pituitary tumors (+1.0 cm; p = 0.06).

**Figure 1:**
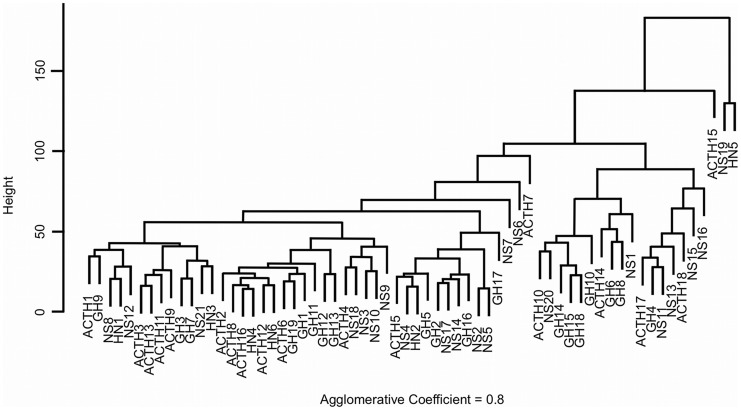
Hierarchical clustering for canonical and non-canonical Wnt pathway genes in normal pituitary and in different subtypes of the pituitary tumors. HN: normal pituitary; ACTH: ACTH-secreting pituitary tumor; GH: GH-secreting pituitary tumor; NS: non-secreting pituitary tumor.

Using the Agnes-algorithm we constructed a hierarchical clustering for canonical and non-canonical Wnt pathway genes in normal pituitary and in pituitary adenomas (Figure 1). The gene expression observed in normal pituitaries and in all subtypes of secreting- and non-secreting pituitary tumors was randomly distributed throughout the dendrogram.

**Figure 2:**
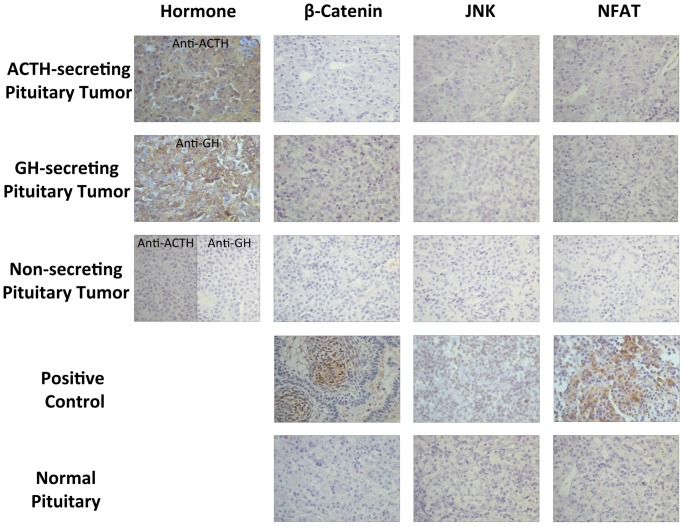
Immunocytochemistry for β-catenin, JNK, and NFAT5 in normal pituitaries, ACTH-secreting pituitary tumor, GH-secreting pituitary tumor, and non-secreting pituitary tumor (x40). ACTH and GH immune positivity are shown in the region of the tumor sample immunostained for β-catenin, JNK and NFAT. Craniopharyngioma tissue and two positive samples from GH-secreting pituitary tumors were used as positive controls for β-catenin, NFAT5, and JNK antibodies, respectively.

At protein level, we evaluated by IHC, three main effectors of canonical (β-catenin), calcium dependent (NFAT5), and planar cell polarity (JNK) of the Wnt pathways. We found no expression of β-catenin protein in all but one normal pituitary, which showed only a weak β-catenin staining (1 to 10% of the cells). The majority of the pituitary tumors also showed no expression of β-catenin; a weak β-catenin staining was observed only in 2 of 16 (12.5%) ACTH-secreting, 1 of 18 (5.5%) GH-secreting, and 1 of 18 (5.5%) non-secreting pituitary tumors. There was no significant difference in the β-catenin staining among all pituitary tumors and normal pituitaries (Figure 2).

We found no expression of JNK protein, the Wnt/polarity pathway effector, in normal pituitaries and a weak staining (less than 10% of the cells) was observed in 12.8%, 5.5%, 5.5% of ACTH-, GH-secreting and non-secreting pituitary tumors, respectively, without difference among pituitary tumors and normal pituitaries (Figure 2). We also found no expression of NFAT protein, the Wnt/calcium pathway effector, in normal pituitaries, GH-secreting, and non-secreting pituitary tumors; but a weak NFAT staining was observed in 18.8% of ACTH-secreting adenomas. There was no significant difference in NFAT staining among all pituitary tumors and normal pituitaries (Figure 2).

## Discussion

The present study assessed the expression of several genes of the canonical and non-canonical Wnt pathways as well as the main protein effectors of these pathways in a subset of ACTH-, GH-secreting and non-secreting pituitary tumors. Our data clearly show that components of the canonical and non-canonical Wnt pathways are not mis-expressed in the pituitary tumors.

Due to sample unavailability, we chose some of the most important genes involved in the different steps of the Wnt pathways that include for canonical: activating ligands (*WNT11, WNT4, WNT5A*), binding inhibitors (*DKK3, sFRP1*), β-catenin gene (*CTNNB1*), β-catenin degradation complex (*APC, AXIN1, GSK3β*), inhibitor of β-catenin degradation complex (*AKT1*), sequester of β-catenin (*CDH1*), pathway effectors (*TCF7, MAPK8, NFAT5*), pathway mediators (*DVL-1, DVL-2, DVL-3, PRICKLE, VANGL1*), and target genes (*MYB, MYC, WISP2, SPRY1, TP53, CCND1*); for calcium pathway: *PLCB1, CAMK2A, PRKCA,* and *CHP*; and for planar cell polarity pathway: *PTK7, DAAM1,* and *RHOA*.

We found some individual variability in the gene expression of canonical and non-canonical Wnt pathways, probably due to individual tumor heterogeneity; which has been frequently observed in arrays from other types of tumors [31]–[34]. No differential expression of the studied genes was observed between normal pituitaries and each different subtype of pituitary adenomas, except for over-expression of *WISP2* in ACTH-secreting pituitary tumor. *WISP2*, a tumor suppressor gene involved in attenuating tumor invasion [35], [36], has a glucocorticoid-responsive region in its promoter [37]. The elevated glucocorticoid levels observed in ACTH-secreting pituitary tumors would, therefore, over activate the *WISP2* transcription.

β-catenin protein expression was also not accumulated in the nucleus/cytoplasm of the pituitary tumor cells, confirming no activation of canonical Wnt pathway in these tumors. The first paper published on β-catenin protein expression in pituitary tumors found β-catenin nuclear accumulation in 21 out of 37 (57%) tumors [15]. However, our data are in agreement with four other studies that found only slight β-catenin staining (0 to 2% of the cells) in a total of 231 pituitary tumors [12], [16], [17]. It is important to point out that *CTNNB1* mutations were not found in 154 pituitary tumors previously evaluated, supporting the lack of the involvement of canonical Wnt pathway in the pathogenesis of pituitary tumors [17]. This is the reason that, in the present study, we did not sequence any gene involved in the Wnt pathway.

In addition, for the first time, we evaluated non-canonical Wnt/calcium and the Wnt/polarity pathways in secreting and non-secreting pituitary tumors. Our data on mRNA and protein expression demonstrate no evidence for the involvement of non-canonical pathways in each different subtype of the pituitary tumors. Previous study had suggested in GHomas and TSHomas a potential activation of non-canonical Wnt/cell polarity pathway, via activation of Erk1/2 MAPK by Wnt4 signaling [38]. However, it is important to note that Erk1/2 signaling may be due to the activation of other pathways, such as mTOR [39] and TGFβ [40].

Furthermore, we observed no association of expression of the studied mRNAs or the three main protein effectors of canonical (β-catenin) and non-canonical Wnt pathways (JNK and NFAT) with tumor size, recurrence, and progressive disease, except for over-expression of *VANGL1,* which was associated to bigger tumors. Indeed, the suppression of *VANGL1* expression has been associated *in vitro* to the inhibition of the hepatocellular carcinoma growth [41]. In addition, *VANGL1* is important in the brain development and mutations in this gene have been associated to neural-tube defects [42]. Finally, we can not exclude some deregulation at other genes or proteins. However, the absence of alterations in the mRNA expression and protein levels of main effectors of canonical (β-catenin), calcium dependent (NFAT5), and planar cell polarity (JNK) suggests that even a hypothetical deregulation of other pathways would not influence directly the expression of genes of the canonical and non-canonical Wnt pathways.

We also used a bioinformatics approach, the Agnes-algorithm, to perform a hierarchical clustering of canonical and non-canonical Wnt pathway genes. The dendrogram showed a regular pattern of genes randomly distributed. We could not find a regular pattern of gene expression that would suggest a deregulated gene expression in any specific subtype of pituitary tumor, confirming the putative no involvement of the Wnt pathways in the pathogenesis of the pituitary tumors.

Recently, an elegant study generates a mouse that expressed a degradation-resistant mutant form of β-catenin in early Rathke's pouch progenitors of the Wnt/β-catenin pathway and demonstrates that β-catenin–accumulating cells formed clusters, which resemble human craniopharyngiomas. These tumors arise from activation of β-catenin in pituitary progenitors during embryogenesis, since these clusters expressed stemness cell markers such as *SOX2*, *SOX9*, and *p27Kip1* [43]. Mutation in the exon 3 of the *CTNNB1* gene that codifies the binding site for the degradation complex of the β-catenin molecule is one of the molecular findings associated with adamantinomatous craniopharyngiomas [11], [12], [16], [44]. Taking together, our data demonstrate no abnormal pattern of cytoplasmatic/nuclear β-catenin distribution in pituitary adenomas in contrast to craniopharyngiomas. From embryogenesis to adulthood, the number of proliferating cells decreases progressively, whereas the number of differentiated cells increases [45]; since there is no activation of Wnt pathways in pituitary tumors, we suggest that these tumors in contrast to craniopharyngiomas might arise from differentiated cells.

In conclusion, our data provide evidence that besides canonical, also the non-canonical Wnt pathway genes are not mis-expressed in the pituitary tumorigenesis.

## Supporting Information

Table S1 **Clinical and Laboratory Features of ACTH-secreting Pituitary Tumors.**
(DOCX)

Table S2 **Clinical and Laboratory Features of GH-secreting Pituitary Tumors.**
(DOCX)

Table S3 **Clinical and Laboratory Features of non-secreting Pituitary Tumors.**
(DOCX)

Table S4 **Genes of canonical and non-canonical Wnt Pathways and the respective qPCR assay identification probes.**
(DOCX)
