# Supplementary material for: Components of the Canonical and Non-Canonical Wnt Pathways Are Not Mis-Expressed in Pituitary Tumors
Source: PLoS One. 2013 Apr 26;8(4):e62424. doi: 10.1371/journal.pone.0062424 (PMC3637156; doi:10.1371/journal.pone.0062424)
Supplement: Table S1 — Clinical and Laboratory Features of ACTH-secreting Pituitary Tumors. (DOCX) [file pone.0062424.s001.docx]

**Supplementary Table S1:** Clinical and Laboratory Features of ACTH-secreting Pituitary Tumors.

| Patients | Age  (years) | Gender | Tumor Size (cm)  (MRI) | IHC |
| --- | --- | --- | --- | --- |
| ACTH 1 | **51** | **F** | **0.5** | **ACTH +** |
| ACTH 2 | **15** | **M** | **No evidence** | **ACTH +** |
| ACTH 3 | **24** | **F** | **0.6** | **ACTH +** |
| ACTH 4 | **12** | **M** | **No evidence** | **ACTH +** |
| ACTH 5 | **27** | **F** | **0.8** | **ACTH +** |
| ACTH 6 | **45** | **F** | **1.0** | **ACTH +** |
| ACTH 7 | **36** | **F** | **1.0** | **ACTH +** |
| ACTH 8 | **31** | **M** | **No evidence** | **ACTH +** |
| ACTH 9 | **15** | **F** | **No evidence** | **ACTH +** |
| ACTH 10 | **54** | **F** | **0.3** | **ACTH +** |
| ACTH 11 | **38** | **F** | **No evidence** | **NA** |
| ACTH 12 | **26** | **F** | **No evidence** | **ACTH +** |
| ACTH 13 | **39** | **F** | **1.6** | **NA** |
| ACTH 14 | **11** | **F** | **0.4** | **ACTH +** |
| ACTH 15 | **19** | **F** | **0.5** | **ACTH +** |
| ACTH 16 | **26** | **F** | **0.8** | **ACTH +** |
| ACTH 17 | **32** | **F** | **0.6** | **ACTH +** |
| ACTH 18 | **17** | **F** | **3.5** | **ACTH +** |

F: female; M: male; MRI: magnetic resonance imaging; IHC: Immunohistochemistry; NA: not available
