# Supplementary material for: Components of the Canonical and Non-Canonical Wnt Pathways Are Not Mis-Expressed in Pituitary Tumors
Source: PLoS One. 2013 Apr 26;8(4):e62424. doi: 10.1371/journal.pone.0062424 (PMC3637156; doi:10.1371/journal.pone.0062424)
Supplement: Table S2 — Clinical and Laboratory Features of GH-secreting Pituitary Tumors. (DOCX) [file pone.0062424.s002.docx]

**Supplementary Table S2:** Clinical and Laboratory Features of GH-secreting Pituitary Tumors.

| Patent | Age (years) | Gender | Tumor Size (cm)(MRI) | IHC | Remission | Visual Field | Basal GH (µg/L) | GH after oGTT (µg/L) | IGF-1  (μg/L) |
| --- | --- | --- | --- | --- | --- | --- | --- | --- | --- |
| GH 1 | 28 | F | 2.4 x 1.5 | GH+, PRL+ | No | Normal | 14.4 | 10.4 | 480 |
| GH 2 | 29 | M | 3.3 x 2.0 | GH+, PRL+ | No | Abnormal | 92.0 | 52.0 | NA |
| GH 3 | 43 | F | 1.0 x 0.8 | GH+, PRL+, LH+ | Yes | Abnormal | 15.5 | 13.5 | 205 |
| GH 4 | 59 | F | 1.0 x 0.9 | GH+, PRL+ | Yes | Normal | 1.7 | 1.7 | NA |
| GH 5 | 34 | M | 1.2 x 1.0 | GH+, PRL+ | No | Normal | 7.1 | 5.4 | 119 |
| GH 6 | 50 | F | 1.2 x 1.0 | GH+ | Yes | Normal | 39.2 | 22.0 | NA |
| GH 7 | 30 | F | 1.3 x 2.0 | GH+ | No | Abnormal | 13.7 | 7.1 | 410 |
| GH 8 | 37 | F | 3.0 x 2.5 | GH+ | No | Normal | 185 | 67 | 297 |
| GH 9 | 57 | M | 2.0 X 1.4 | GH+ | No | Abnormal | 47.8 | 28.9 | 758 |
| GH 10 | 39 | M | 2.5 x 2.7 | GH+, PRL+ | No | Abnormal | 81.0 | 89 | 360 |
| GH 11 | 52 | F | 3.3 x 3.4 | GH+, PRL+, LH+ | No | Normal | 23.0 | 19.5 | 376 |
| GH 12 | 32 | M | 3.8 x 2.6 | GH+, TSH+, PRL+, LH+ | No | Abnormal | 10.8 | 10.8 | NA |
| GH 13 | 56 | F | 1.8 x 1.3 | GH+ | No | Normal | 1.6 | 12.0 | NA |
| GH 14 | 53 | F | 2.5 x 1.8 | GH+ | No | Normal | 20.9 | 19.2 | 540 |
| GH 15 | 43 | M | 0.9 x 0.7 | GH+ | Yes | Normal | 4.9 | 6.3 | 2060 |
| GH 16 | 37 | F | 2.9 x 1.9 | GH+, PRL+ | No | Abnormal | 10.0 | 8.2 | 735 |
| GH 17 | 42 | M | 2.3 x 1.5 | GH+, TSH+, PRL+ | No | Normal | 110.0 | 112.0 | NA |
| GH 18 | 31 | F | 1.3 x 1.1 | GH+, PRL+, TSH+ | No | Normal | 119.0 | 130.0 | NA |
| GH 19 | 24 | M | 5.0 x 3.0 | GH+, TSH+ PRL+ | No | Abnormal | 392.5 | 428.0 | NA |

F: female; M: male; MRI: magnetic resonance imaging; IHC: Immunohistochemistry; NA: not available; oGTT: oral glucose tolerance test
