# Supplementary material for: Components of the Canonical and Non-Canonical Wnt Pathways Are Not Mis-Expressed in Pituitary Tumors
Source: PLoS One. 2013 Apr 26;8(4):e62424. doi: 10.1371/journal.pone.0062424 (PMC3637156; doi:10.1371/journal.pone.0062424)
Supplement: Table S3 — Clinical and Laboratory Features of non-secreting Pituitary Tumors. (DOCX) [file pone.0062424.s003.docx]

**Supplementary Table 3:** Clinical and Laboratory Features of non-secreting Pituitary Tumors.

| Patient | Age  (years) | Gender | Tumor Size (cm)  (MRI) | IHC | Remission | Visual Field |
| --- | --- | --- | --- | --- | --- | --- |
| NS 1 | 61 | M | 1.9 x 2.0 | Negative | Yes | Normal |
| NS 2 | 45 | M | 2.4 x 1.9 | PRL+, LH+, FSH+ | No | Normal |
| NS 3 | 37 | M | 7.7 x 4.4 | LH+, FSH+ | No | Abnormal |
| NS 4 | 42 | F | 3.2 x 3.0 | LH+ | Yes | Abnormal |
| NS 5 | 43 | M | 4.3 x 3.7 | Negative | Yes | Abnormal |
| NS 6 | 42 | F | 3.5 x 2.8 | Negative | Yes | Abnormal |
| NS 7 | 70 | M | 4.1 x 3.2 | Negative | No | Abnormal |
| NS 8 | 50 | M | 3.0 x 4.0 | LH+ | Yes | Abnormal |
| NS 9 | 58 | M | 4.1 x 2.5 | Negative | Yes | Abnormal |
| NS 10 | 64 | F | 2.7 x 2.0 | LH+ | Yes | Normal |
| NS 11 | 71 | M | 2.9 x 1.8 | TSH+, LH+, FSH+ | Yes | Abnormal |
| NS 12 | 64 | M | 3.0 x 2.6 | LH+ | No | Abnormal |
| NS 13 | 31 | F | 2.9 x 3.0 | Negative | Yes | Abnormal |
| NS 14 | 47 | F | 2.0 x 1.6 | Negative | Yes | Abnormal |
| NS 15 | 49 | M | 2.1 x 2.4 | TSH+ | Yes | Abnormal |
| NS 16 | 40 | F | 2.5 x 1.9 | Negative | Yes | Abnormal |
| NS 17 | 27 | F | 5.1 x 4.4 | Negative | No | Abnormal |
| NS 18 | 27 | M | 2.3 x 2.0 | Negative | Yes | Normal |
| NS 19 | 64 | F | 4.0 x 2.7 | LH+ | Yes | Abnormal |
| NS 20 | 17 | F | 1.0 x 1.0 | Negative | No | Normal |
| NS 21 | 50 | M | 4.2 x 2.5 | PRL+, LH+, TSH+ | No | Abnormal |

F: female; M: male; MRI: magnetic resonance imaging; IHC: Immunohistochemistry;
