# Supplementary material for: Components of the Canonical and Non-Canonical Wnt Pathways Are Not Mis-Expressed in Pituitary Tumors
Source: PLoS One. 2013 Apr 26;8(4):e62424. doi: 10.1371/journal.pone.0062424 (PMC3637156; doi:10.1371/journal.pone.0062424)
Supplement: Table S4 — Genes of canonical and non-canonical Wnt Pathways and the respective qPCR assay identification probes. (DOCX) [file pone.0062424.s004.docx]

**Supplementary Table S4:** Genes of canonical and non-canonical Wnt Pathways and the respective qPCR assay identification probes.

| **Gene** | **Assay ID** |
| --- | --- |
| ***CTNNB1*** | Hs00170025_m1 |
| ***WNT11*** | Hs00182986_m1 |
| ***WNT4*** | Hs00229142_m1 |
| ***WNT5A*** | Hs00998537_m1 |
| ***DKK3*** | Hs00247426_m1 |
| ***sFRP1*** | Hs00610060_m1 |
| ***APC*** | Hs01568270_m1 |
| ***AXIN1*** | Hs00394718_m1 |
| ***GSK3β*** | Hs00275656_m1 |
| ***AKT1*** | Hs00178289_m1 |
| ***CDH1*** | Hs01023895_m1 |
| ***TCF7*** | Hs00175273_m1 |
| ***MAPK8*** | Hs00177083_m1 |
| ***NFAT5*** | Hs00232437_m1 |
| ***DVL-1*** | Hs00182896_m1 |
| ***DVL-2*** | Hs00182901_m1 |
| ***DVL-3*** | Hs00610263_m1 |
| ***PRICKLE*** | Hs01055551_m1 |
| ***VANGL1*** | Hs00371144_m1 |
| ***PLCB1*** | Hs00248563_m1 |
| ***CAMK2A*** | Hs00392405_m1 |
| ***PRKCA*** | Hs00925195_m1 |
| ***CHP*** | Hs00819122_m1 |
| ***PTK7*** | Hs00177173_m1 |
| ***DAAM1*** | Hs00323674_m1 |
| ***RHOA*** | Hs00357608_m1 |
| ***MYB*** | Hs00920556_m1 |
| ***MYC*** | Hs99999003_m1 |
| ***WISP2*** | Hs00180242_m1 |
| ***SPRY1*** | Hs00398096_m1 |
| ***TP53*** | Hs01034249_m1 |
| ***GUSβ*** | Hs00939627_m1 |
| ***TBP*** | Hs00427621_m1 |
| ***PGK1*** | Hs99999906_m1 |

qPCR: real time polymerase chain reaction. Probes were obteined by TaqMan® Real Time PCR Assay (Applied Biosystems, Foster City, CA, USA)
